# Paroxysmal Hemidystonia as the Presenting Manifestation of Previously Undiagnosed Diabetes Mellitus

**DOI:** 10.5334/tohm.1185

**Published:** 2026-03-24

**Authors:** Subhankar Chatterjee, Samya Sengupta, Ritwik Ghosh, Julián Benito-León, Souvik Dubey

**Affiliations:** 1 Department of Endocrinology & Metabolism, Medical College & Hospital, Kolkata, India; 2 Department of Neuromedicine, Bangur Institute of Neurosciences, IPGMER & SSKM Hospital, Kolkata, India; 3 Department of General Medicine, Burdwan Medical College & Hospital, Burdwan, India; 4 Department of Neurology, 12 de Octubre University Hospital, Madrid, Spain; 5 Instituto de Investigación Sanitaria Hospital 12 de Octubre (imas12), Madrid, Spain; 6 Centro de Investigación Biomédica en Red Sobre Enfermedades Neurodegenerativas (CIBERNED), Madrid, Spain; 7 Department of Medicine, Complutense University, Madrid, Spain

**Keywords:** paroxysmal hemidystonia, hyperglycemia, diabetes mellitus, diabetic striatopathy, 18F-FDG PET/CT

## Abstract

**Background:**

Hyperglycemia-related movement disorders classically present as hemichorea-hemiballismus, whereas dystonia is far less common and may occasionally precede the recognition of diabetes mellitus.

**Case Report:**

A 72-year-old man presented with a 3-month history of recurrent, stereotyped dystonic spells involving the right side of the face, arm, and leg. These episodes occurred approximately twice per minute and lasted about 5 seconds each. Initial laboratory testing revealed a plasma glucose level of 494 mg/dL and an HbA1c of 16.1%, consistent with previously unrecognized diabetes mellitus. Electrolyte levels, electroencephalography, cerebrospinal fluid analysis, and evaluation for autoimmune encephalopathy were unremarkable. Brain MRI showed only a chronic lacunar infarct in the left basal ganglia, with no evidence of an acute striatal lesion. The abnormal movements resolved completely within 3 days after correction of hyperglycemia. Follow-up 18F-FDG PET/CT performed after clinical recovery demonstrated subtle relative hypometabolism in the left cerebellum, with preserved uptake in the basal ganglia and cerebral cortex.

**Discussion:**

This case broadens the phenomenologic spectrum of hyperglycemia-related movement disorders and highlights paroxysmal hemidystonia as a potential presenting manifestation of previously undiagnosed diabetes mellitus. Bedside glucose testing should therefore be routinely performed in the evaluation of new-onset paroxysmal hyperkinetic movements. In addition, isolated extra-striatal metabolic abnormalities detected after symptom resolution should be interpreted with caution.

Hyperglycemia-induced involuntary movements are a well-recognized and potentially reversible neurological complication of severe hyperglycemia. Hemichorea-hemiballismus is the prototypical presentation and is often accompanied by contralateral striatal abnormalities on magnetic resonance imaging (MRI) or computed tomography (CT), forming the clinicoradiological syndrome known as diabetic striatopathy (DS) [1,2]. Non-choreic presentations, including dystonia and mixed hyperkinetic phenomenology, are less commonly reported but are clinically important because abnormal movements may precede the diagnosis of diabetes mellitus and remit rapidly after correction of hyperglycemia [3,4,5,6,7].

We report a 72-year-old man with a three-month history of recurrent, stereotyped episodes of abnormal involuntary movements involving the right face, arm, and leg (approximately two episodes per minute, each lasting about 5 seconds; Video 1). The events consisted of slow, jerky, twisting movements culminating in a brief sustained torsional posture, consistent with a dystonic phenomenology (Video 1). Between attacks, the neurological examination was normal. Initial laboratory testing demonstrated marked hyperglycemia (plasma glucose 494 mg/dL) and an HbA1c of 16.1%, indicating previously unrecognized diabetes mellitus. Serum electrolytes were within normal limits, including sodium 140 mmol/L, potassium 4.2 mmol/L, and total calcium 9.8 mg/dL. Levetiracetam (500 mg twice daily), prescribed elsewhere, was ineffective and was discontinued. Glycemic control was achieved with oral glucose-lowering therapy, and the paroxysmal movements resolved completely within three days as the plasma glucose fell to 180 mg/dL. Brain MRI showed only a small chronic lacunar infarct in the left basal ganglia and minimal chronic white-matter changes (Figure 1A). Electroencephalography, cerebrospinal fluid analysis, and an autoimmune encephalopathy panel were unremarkable. One month later, after complete clinical resolution and with stable glycemia (plasma glucose 136 mg/dL), brain 18F-fluorodeoxyglucose positron emission tomography/computed tomography (18F-FDG PET/CT) showed subtle asymmetric relative hypometabolism confined to the cerebellum, more evident in the left cerebellar hemisphere, with preserved uptake in the basal ganglia and cerebral cortex (Figure 1B).

**Video 1:** Recurrent stereotyped right hemidystonic paroxysms involving the right side of the face, arm, and leg, occurring approximately twice per minute, each lasting around 5 seconds, with twisting movements and a brief sustained abnormal torsional posture, consistent with hemidystonia.

**Figure 1:**
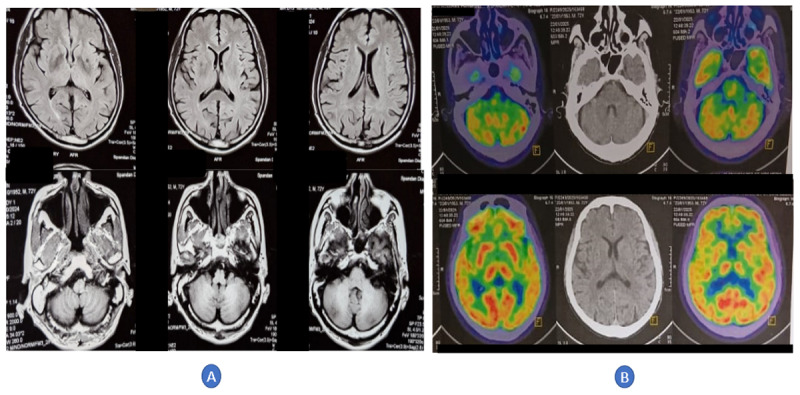
Structural MRI and 18F-FDG PET/CT findings. **(A)** Axial brain MRI shows a small chronic lacunar infarct in the left basal ganglia, minimal periventricular white-matter change, and age-appropriate cortical atrophy. **(B)** Brain 18F-FDG PET/CT shows mild asymmetric relative cerebellar hypometabolism, more evident in the left cerebellar hemisphere, with preserved uptake in the basal ganglia and cerebral cortex.

Phenomenologically, the combination of stereotypy, twisting quality, and brief sustained abnormal posturing supports the diagnosis of paroxysmal hemidystonia. This places our case alongside prior reports in which hemidystonia, focal dystonia, or choreo-dystonia served as an early clue to diabetes or severe hyperglycemia [3,4,5,6,7,8,9]. Together, these reports highlight a practical point: in older adults presenting with new-onset paroxysmal hyperkinetic movements, bedside glucose testing can prevent misdiagnosis and unnecessary treatment [1,2]. Although rapid improvement with normalization of glycemia is typical, persistence has occasionally been described in patients with hemichorea-hemidystonia [8].

In DS, striatal T1 hyperintensity or CT hyperdensity is common, and 18F-FDG PET studies have demonstrated reduced glucose metabolism in the affected basal ganglia, supporting regional metabolic failure [1,2,10,11]. Our patient had no acute striatal lesion on MRI; however, this does not exclude basal ganglia dysfunction, because clinically isolated DS may occur with normal structural imaging [10]. The chronic lacunar infarct in the left basal ganglia may have rendered the ipsilateral striatal network vulnerable (a “first hit”), lowering the threshold for involuntary movements when severe hyperglycemia supervened (a “second hit”) [12,13]. This vascular-metabolic framework is consistent with the proposed overlap between stroke and diabetic striatopathy, in which endothelial dysfunction, atherosclerotic disease, diabetic microangiopathy, and hyperosmolarity may converge to impair regional perfusion and metabolism [2,13]. In this context, right-sided hemidystonia together with a chronic left basal ganglia lacune may reflect a shared substrate rather than a simple incidental finding. Nevertheless, this interpretation remains speculative because the lesion was old, no acute striatal abnormality was seen, and the movements resolved rapidly after glycemic correction.

The mild asymmetric cerebellar hypometabolism on 18F-FDG PET/CT provides an additional, nonspecific imaging observation. Because the PET/CT was obtained after complete clinical resolution, the asymmetry was subtle, there were no cerebellar signs, and basal ganglia uptake was preserved, we do not infer a causal role. Although contemporary models of dystonia emphasize distributed motor circuits connecting the cerebellum, basal ganglia, thalamus, and cortex, a single post-resolution cerebellar metabolic asymmetry is best regarded as hypothesis-generating and may represent an incidental, downstream, or compensatory network effect rather than the primary driver of symptoms [14].

In summary, this case highlights paroxysmal hemidystonia as the presenting manifestation of previously undiagnosed diabetes mellitus, with rapid and complete resolution after glycemic correction. Bedside glucose testing should be routine in assessing new-onset paroxysmal hyperkinetic movements. When functional imaging reveals extra-striatal abnormalities, interpretation should take into account the timing of imaging relative to symptoms and the possibility of nonspecific network effects.
